# Understanding pharmaceutical industry coalitions: a case study of Mallinckrodt

**DOI:** 10.1057/s41599-026-08961-8

**Published:** 2026-09-22

**Authors:** Kgosi Tavares, Tanner Wakefield, Eric Crosbie, Dorie E. Apollonio

**Affiliations:** 1 University of Nevada, Las Vegas, NV, USA; 2 University of California, San Francisco, CA, USA

## Abstract

The opioid industry has used coalitions to lobby for policies beneficial to its interests and to influence public discourse. In this study, we reviewed the creation of Mallinckrodt’s Alliance for Balanced Pain Management (AfBPM) to better understand opioid industry coalitions. We searched the Opioid Industry Documents Archive (OIDA), an online, open-access collection of documents released in US litigation against opioid manufacturers, for materials on AfBPM. We conducted a retrospective qualitative content analysis of 132 documents, including emails, press releases, and internal presentations, by coding them by themes drawn from the Policy Dystopia model. In the mid-2010s, Mallinckrodt created AfBPM to position itself as a responsible opioid manufacturer. It recruited independent organizations and held a 2014 summit to draw press attention. It sought to convey the message that opioid misuse was the responsibility of healthcare providers and patients, and used its website, news media campaigns, op-eds, and social media to spread these messages. Mallinckrodt used AfBPM as a tool to recruit other organizations and to claim that patients and prescribers were responsible for opioid misuse. Understanding industry use of coalitions like AfBPM can help develop policies that prevent healthcare-focused coalitions from being co-opted by pharmaceutical manufacturers.

## Introduction

Pharmaceutical marketing practices are a scientifically documented mechanism by which commercial interests can influence the practice of medicine (Saavedra, O’Connor and Fugh-Berman, 2017; Ortiz and Rosenthal, 2019; Schwartz and Woloshin, 2019; Kirubarajan et al., 2023). Beginning in the 1990s, US pharmaceutical companies marketed prescription opioids as non-addictive when used for pain and encouraged increased physician prescribing, leading to widespread overuse, misuse, and deaths (Drug Topics, 2021; Yakubi et al., 2022). This marketing resulted in a peak dispensing rate of 81.3 prescriptions per 100 Americans in 2012 (Hill et al., 2008; Ross et al., 2008; Landefeld and Steinman, 2009; Jørgensen, Jørgensen and Gøtzsche, 2010; Vedula et al., 2012; U.S. Opioid Dispensing Rate Maps, 2021; Apollonio, 2022). Previous studies of the opioid epidemic suggest relevant hypotheses on how opioid manufacturers successfully influenced clinicians, patients, and regulators through targeted marketing, interference in scientific research on opioid safety, lobbying for weak state regulations, and exploitation of stigmatizing assumptions about addiction (Chisholm-Burns et al., 2019; DeWeerdt, 2019; Merino et al., 2019; Salmond and Allread, 2019; Wood and Elliott, 2020).

A large body of research using tobacco industry documents demonstrates why and how companies misrepresented research to claim that tobacco was not addictive (Glantz et al., 1995; Bero, 2003; Apollonio and Malone, 2005; Cataldo and Malone, 2008; Prochaska, Hall and Bero, 2008), resulting in comprehensive state and federal tobacco control policies (Hurt and Robertson, 1998; Bero, 2003; Elias, Hendlin and Ling, 2018). Past research has found that pharmaceutical companies used similar strategies to sell opioids,(Alexander, Mix and Choudhury, 2022) including targeted marketing to vulnerable populations (Yakubi et al., 2020a; Yakubi et al., 2022), misrepresenting research on the addictive potential of prescription opioids and the risk of overdose, (White and Bero, 2010; RD et al., 2018; Gac et al., 2020; Yakubi et al., 2020b, 2020a) and lobbying to block, weaken, or delay regulatory efforts to control opioid prescribing (DeLeo and Duarte, 2021). Recent research has called for a better understanding of how actors such as pharmaceutical companies seek to influence regulation and public opinion, given that, unlike tobacco, their products (Alexander et al., 2022; The Commercial Determinants of Health, 2022; The Lancet, 2023).

In this study, we sought to address this gap in research on pharmaceutical industry activities affecting public health by assessing opioid manufacturers’ efforts to influence public opinion and to deter stricter government regulation in the mid-2010s. This period followed the rapid increase in opioid-related deaths that began in 1999 (Understanding the Opioid Overdose Epidemic, 2024). Our objective was to understand the opioid industry’s approach to public relations, and to what extent its stated goals to advocate on behalf of people in pain were related to profit-seeking and image management. Additionally, we compared the activities of the opioid industry with past interference efforts by the tobacco industry to determine whether there are shared anti-regulatory tactics across market sectors. We considered these questions through a case study of the Alliance for Balanced Pain Management (AfBPM), which was established in 2014 by Mallinckrodt, a pharmaceutical company focused on generic drug manufacturing and sales that was later found to have played a central role in creating the US opioid crisis (Davis, Boburg and O’Harrow Jr., 2019). The move to create the AfBPM formed amid federal regulatory scrutiny of Mallinckrodt, which began in 2011 after the Drug Enforcement Administration (DEA) found the company’s pills in large, illegal shipments by its partner drug distributors (Bernstein and Higham, 2017). The government believed nearly 44,000 orders that Mallinckrodt fulfilled from retailers and distributors were suspicious (Bernstein and Higham, 2017). Mallinckrodt sold 2.3 billion opioid pills between 2006 and 2014, and was one of the largest producers of opioid medications in the United States. The company announced to shareholders that it was under federal investigation in 2014 (Bernstein and Higham, 2017), the same year it established AfBPM. Our goal was to explore how alliances with external organizations were formed, assess the extent of companies’ influence over alliance membership and decision-making, and identify the methods industry-sponsored coalitions used to influence policy and public opinion.

## Methods

Industry documents research is a key strategy used by public health researchers investigating corporate political activity to understand commercial determinants of health (CDC, 2024; Opioid Industry Documents, no date; “Overview - Opioids Industry Documents”, no date). We conducted a retrospective qualitative review of pharmaceutical industry documents released in litigation and available in the Opioid Industry Documents Archive (OIDA). OIDA was created through a partnership between the University of California, San Francisco (UCSF) and Johns Hopkins University (JHU) (“Overview - Opioids Industry Documents”, no date). Ethical approval and informed consent were not required for this study, as it did not involve human participants and relied solely on publicly available records (UCSF IRB #10-01262). As of September 2025, OIDA contained 5,402,199 internal corporate documents released in opioid litigation, including email communications, advertisements, marketing materials, submissions to regulatory agencies, and prescribing publications (Opioid Industry Documents, no date). OIDA was created to generate new knowledge that could identify structural factors underlying the opioid epidemic and motivate changes in public policy as well as public health practices to prevent future harms (“Overview - Opioids Industry Documents”, no date).

Methods for industry documents analysis were first developed and validated by tobacco researchers (Bero, 2003), but have more recently been employed to examine opioid industry behavior (Chiu et al., 2023; Gac et al., 2024; Gupta et al., 2024; Haddock et al., 2025; Kabella et al., 2025). Following the practices established by prior research on industry documents on tobacco and opioids, we conducted a retrospective review of documents assessing industry interference activities using the Policy Dystopia Model. We conducted an initial search using the keywords “marketing,” “Mallinckrodt,” and “safe” between November 2024 and March 2025, selecting those terms to focus on an issue related to the opioid crisis and industry regulatory concerns, and to limit our findings to one company. After this preliminary keyword search, additional documents were identified using a snowball search, incorporating new search terms such as the names of organizations and initiatives mentioned in the documents (e.g., “government affairs”). Of an initial 6635 results, 134 documents were identified as relevant for further review by an author with 10 years of industry documents research experience; documents identified as irrelevant included duplicates, scheduling emails, blank pages, or material that could not be identified as relating to the events covered (see Supplement for details). Two reviewers independently coded these documents and organized them by themes identified in the Policy Dystopia Model, a taxonomy of activities employed by the alcohol and tobacco industries to interfere with regulation (Ulucanlar, Fooks and Gilmore, 2016). Developed through a review of peer-reviewed research on alcohol and tobacco companies’ anti-regulatory efforts, the Policy Dystopia Model is a behavioral framework that classifies industry tactics into discursive (rhetorical) and instrumental (action-based) activities (Ulucanlar, Fooks and Gilmore, 2016). Discursive strategies refer to coalition development and/or deployment to influence policy, challenging public health messaging, disseminating industry information, and using direct tactics to sway policy. Instrumental strategies involve framing messages around societal and economic costs and unintended negative impacts (harmful to public health or benefiting individuals, such as illegal sellers) (Ulucanlar, Fooks and Gilmore, 2016). We coded documents using the discursive and instrumental strategies established by the policy dystopia model to assess strategic and behavioral similarities between the opioid and tobacco industries. If the reviewers disagreed on a classification, they discussed it until a coding decision was reached. These industry documents were categorized as follows: 63 general documents, 69 emails, and 13 presentations. Documents were then arranged in chronological order. Document dates ranged from 2014 to 2017. Of the 134 documents, the 25 that were most comprehensive (providing the most detail) and relevant to the AfBPM project and its activities were included and cited in the manuscript. Our initial search began broadly with generic terms, yielding a large body of potentially relevant documents. A review of these documents led to the inclusion of additional keywords: “AfBPM” and “Alliance for Balanced Pain Management”. The discovery and prioritization of these terms substantially reduced the number of relevant documents, as did the elimination of redundant, duplicate, or otherwise meaningless documents (e.g., some documents reference presentations or written works that do not exist in the archive).

Materials in the Opioid Industry Documents Archive are drawn from litigation and do not represent a comprehensive record; while the archive consists of millions of documents, there may be pertinent records that were not part of discovery or release agreements tied to settlements, are considered privileged despite their production in litigation, or simply went undiscovered during our investigation due to the sheer volume of information. In addition, authors of industry documents may refer to planned activities that do not materialize. The nature of the Opioid Industry Documents Archive means our analysis may miss data or capture discussion of plans that were not implemented. Despite these limitations, internal industry documents offer a unique window into the plans and intentions of corporate actors. As a validation strategy, we triangulated our findings with outside professional news and corporate sources. Future research may verify and build on these findings as additional documents are released to the public.

## Results

Our results are organized chronologically and consider three time periods: (1) AfBPM formation, (2) summit planning, and (3) post-summit activities. Using themes identified in the policy dystopia model, these documents were further categorized by (a) coalition management (constituency recruitment (external/internal), and (b) information management (production, amplification, credibility, and reputation management). Our analysis considers and compares activities by AfBPM and Mallinckrodt in the context of the Policy Dystopia Model’s discursive and instrumental behavior taxonomies. That said, limiting this analysis to Mallinckrodt’s efforts with one coalition narrows the scope of behaviors we captured that relate to the Policy Dystopia Model primarily to coalition, information dissemination, and reputation enhancement tactics outlined by the model. A broader analysis of Mallinckrodt’s activities is needed to determine the Model’s total applicability.

### AfBPM formation

In 2014, the Government Affairs division of Mallinckrodt Pharmaceuticals created an Advocacy department “with the objective of elevating Mallinckrodt’s Leadership Role as a responsible manufacturer committed to innovative solutions to address patient needs” (No Author, 2014g). In internal documents, Mallinckrodt stated that it wanted to demonstrate its commitment to safe use and that it needed to “demonstrate how we are a different company than other opioid manufacturers” (No Author, 2014h).

Mallinckrodt’s Advocacy department was the founding sponsor of the Alliance for Balanced Pain Management (AfBPM), a coalition of pharmaceutical industry stakeholders: health care advocacy groups, patient organizations, and industry representatives (No Author, 2014a). Kevin Webb, the Director of Advocacy Relations at Mallinckrodt, played a vital role in the creation and management of AfBPM (Koze and Anderson, 2014). Mallinckrodt, whose subsidiary SpecGx supplied a major share of generic opioids sold in the US by the 2000s (“Collections - Opioids Industry Documents,” no date), established the group as an advocacy coalition (Ulucanlar, Fooks and Gilmore, 2016) (No Author, 2014g). An internal Mallinckrodt presentation from November 2014 described the AfBPM as an organization advocating for “an integrated, balanced approach to pain management and an additional focus on the safe, appropriate use and disposal of opioids” (No Author, 2014b). Mallinckrodt created AfBPM to drive this narrative, claiming the coalition would develop public education campaigns around these advocacy goals [the nature of these campaigns was not described] (No Author, 2014g). Mallinckrodt anticipated that AfBPM would conduct media outreach through appearances and speeches and engage in 2–3 unspecified “action-oriented” initiatives (No Author, 2014g).

Mallinckrodt created the AfBPM to further its mission to “represent the rights of people with pain to receive appropriate pain management while striving for restoration of function balanced with misuse deterrence” (No Author, 2014g). In October 2014, Mallinckrodt acknowledged that “[our] corporate reputation has been greatly enhanced through awareness with key stakeholders” (No Author, 2014h). The decision to recruit external parties to form an advocacy coalition mirrors tactics outlined in the Policy Dystopia Model (Ulucanlar et al., 2016).

Under the Memorandum of Understanding for AfBPM, signed in October 2014, the coalition was structured into a Steering Committee, general alliance members, and the Alliance convener, Mallinckrodt (No Author, 2014i). Leadership of the Alliance was the responsibility of the Steering Committee, which included Mallinckrodt Pharmaceuticals, the Alliance for Patient Access, the American Academy of Physical Medicine & Rehabilitation, the American Cancer Society, the American Chronic Pain Association, the Partnership for Drug-Free Kids, and the Gerontological Society of America (No Author, 2014b). According to a Mallinckrodt organizational chart of AfBPM, the company planned to establish the Steering Committee starting in April 2014 and, by October 2014, recruit additional stakeholders involved in opioids (No Author, 2014b). These stakeholders included patient advocacy organizations, policymakers, pharmacies, the pharmaceutical industry, law enforcement, and professional organizations (No Author, 2014b). The recruited stakeholders were expected to lend their names to public awareness communications created by AfBPM and to incorporate the coalition’s messaging within their own organizations (No Author, 2014i). The Steering Committee was responsible for establishing initiatives, reviewing media coverage of AfBPM, approving new members, and assessing the coalition’s mission.(No Author, 2014i)

Mallinckrodt hired Green Room Communications to help form and manage AfBPM (Koze and Anderson, 2014). Green Room Communications was described by Stephanie Koze, its senior director of client service and strategy, as a healthcare communications firm that supported Mallinckrodt (Koze, 2014a). The stated objective of AfBPM was to foster public dialog about access of pain patients to pain management, as well as responsible patient use, storage, and disposal of these medications (No Author, 2014e). The coalition scheduled its launch for November 18–19, 2014, with an inaugural summit, press release, pitch emails to recruit additional organizations, social media activities, and the launch of a website (No Author, 2014e). Green Room Communications was responsible for planning the summit meeting, facilitating presentations, and managing logistics (No Author, 2014e).

### Launch of the alliance for balanced pain management (AfBPM)

The launch of AfBPM took place from November to December 2014 (No Author, 2014e). It began with an inaugural summit, which took place on November 18th and 19th, 2014, in Washington, DC (No Author, 2014d). On the first day of the summit, Alliance Steering Committee member Rebecca Kirch, Director of Quality of Life and Survivorship for Cancer Control at the American Cancer Society, introduced the objectives and members of the coalition (No Author, 2014j, 2014d). Mary Bono, a former US Member of Congress, coordinated their efforts for balanced pain management and provided tips about working with disparate groups (No Author, 2014d). Knox Todd, MD, from the MD Anderson Cancer Center in Texas, presented on interdisciplinary pain management and the role AfBPM could play. The US Senate Homeland Security and Governmental Affairs Committee report ‘Fueling an Epidemic’ later noted that Todd had received substantial funding from opioid manufacturers (No Author, 2014j). Both of these speeches aligned with Mallinckrodt’s goal of incorporating its expanded portfolio of “intrathecal, IV APAP, Autoimmune & Rare Diseases” products into the summit (No Author, 2014h).

Following these speeches, AfBPM (supported by Green Room) organized workshops to brainstorm initiatives and tactics around balanced pain management (No Author, 2014d). The workshops were centered on “how to help ensure that people with chronic or acute pain have access to appropriate pain management”, and “how to help ensure that patients and providers are educated and informed about responsible prescription, dispensing, use, storage, and disposal of pain medications.” These workshops positioned access to medicines as a public problem, and overuse and addiction as individual responsibilities that were unrelated to corporate marketing of these medications (No Author, 2014d).

The second day of the summit began with members discussing ideas generated during their workshops and proposing activities to increase access and responsible medication management (No Author, 2014d). They suggested gathering patient and provider perspectives about the needs and wants of pain patients and creating a checklist to assess vulnerabilities and solutions for pain patients (No Author, 2014d). Additionally, AfBPM participants proposed focusing on educating medical professionals about the “Screening and Brief Intervention” code, which would allow them to be reimbursed for assessing the risk of substance use disorder and recommending services (No Author, 2014d).

The AfBPM website release, along with a media campaign that included the summit press release, spokesperson training, pitch emails, and a Q&A document, occurred on November 20th, 2014.(No Author, 2014e, 2014d). AfBPM also planned to develop op-eds, education campaigns, and other content focused on appropriate access to and responsible use of opioids (No Author, 2014b). AfBPM prepared a spokesperson document for media interviews as part of their media outreach plan (No Author, 2014e, 2015a). The Q&A focused on balanced pain management and proposed that a “multi-pronged” approach was more effective than any single treatment. This statement aligned with Mallinckrodt’s promotion of multimodal analgesia (MMA), which combined opioid and non-opioid medications, both of which were manufactured by the company (No Author, 2015a; News Detail ∣ Mallinckrodt Pharmaceuticals, no date). Mallinckrodt presentation slides stated that the company supported multimodal analgesia because “[it] care[s] about patient safety” and that “Mallinckrodt provide[s] products along the pain continuum” (No Author, 2024b). The Alliance also planned to develop op-eds, educational campaigns, and other content around appropriate access and responsible use of opioids (No Author, 2014b, 2024b). AfBPM objectives centered on the Policy Dystopia Model’s instrumental tactic of widely circulating the industry’s preferred information (Ulucanlar et al., 2016).

The Green Room team had begun developing AfBPM’s website in September 2014 (Koze, 2014b). The goal of the website was to “foster ongoing engagement and drive repeat visits through Alliance and its member organizations” and to “…aggregate alliance member and for a widespread audience (e.g., physicians, pharmacists, caregivers/consumers, etc.)” (No Author, 2014e). The website’s education section defined “balanced pain management” as “… an approach to treating, improving and controlling pain that uses a combination of approaches…combined with a commitment to safely use, store and dispose of pain medication” (No Author, 2014f). This messaging aligned with a different Mallinckrodt program, which involved the distribution of personal drug disposal pouches, known as Medsaway pouches, that “deactivated” controlled substances and rendered them useless, and were promoted as a way to reduce opioid diversion (No Author, no date). Medsaway pouches were manufactured by Verde Environmental Technologies, with which Mallinckrodt partnered to distribute its product starting in 2014. Medsaway pouch dissemination reflected the Policy Dystopia Model tactic of image rehabilitation (Ulucanlar et al., 2016); In 2014, Mallinckrodt described Medsaway as having strengthened Mallinckrodt’s reputation “as an organization committed to addressing opioid misuse and diversion” (No Author, 2014h, 2014c).

### Post-launch activities

Following the inaugural AfBPM summit, Mallinckrodt proposed developing an “ongoing news engine to establish Alliance as [a] go-to resource for balanced pain management” (No Author, 2014e). The AfBPM wanted to focus on media, newspapers, national and local consumer outlets, television, radio, and social media (No Author, 2014e). Mallinckrodt’s goal was to publicize the idea of balanced pain management as well as safe use, prescription, and disposal of prescription opioid medications (No Author, 2014e). An Alliance for Balanced Pain Management white paper produced in May 2016 echoed these messaging objectives while inferring that restricting opioid prescriptions was not feasible, stating, “…the Centers for Disease Control and Prevention issued their first-ever guidelines for dispensing opioids, recommending physicians to use caution when prescribing them for chronic pain. Yet not enough alternatives to opioids are available to prescribers and their patients to effectively and comprehensively treat pain through other means” (Alliance for Balanced Pain Management, 2016). AfBPM also indicated in 2015 that it would expand its mission to include MMA (Green Room Communications, 2015).

AfBPM began structuring working groups on November 26, 2014, and hoped to recruit additional organizations, including the American Academy of Family Physicians, other professional medical organizations, and the AARP (No Author, 2014c). In April 2016, the AfBPM website listed none of these organizations [we were unable to determine why those organizations did not participate]; however, they were able to recruit other organizations, including the American Academy of Pain Management, American Society for Pain Management Nursing, and the National Alliance of State Pharmacy Associations (No Author, 2016a). Mallinckrodt later approved $25,000 in unspecified grant funding to the American Academy of Pain Management (AAPM) during March 2015, but we found no evidence that the funding was related to AAPM’s participation in AfBPM (Elsbernd and Lassiter, 2015). The documents released to OIDA also did not indicate there were financial relationships between Mallinckrodt and the American Society for Pain Management Nursing or the National Alliance of State Pharmacy Associations. The two working groups from the summit continued to meet; the first focused on access to appropriate pain management, and the second on responsible prescribing, dispensing, use, storage, and disposal; Mallinckrodt was involved in the latter as one of multiple “champion” working group members, meaning it could spearhead parts of monthly calls and in general serve as one of its subject matter experts (No Author, 2016a). The second group was responsible for creating a checklist to assess vulnerabilities and provide solutions, which would be provided through the internet or a brochure (No Author, 2014c).

Mallinckrodt considered capitalizing on the major motion picture CAKE, which was widely released in early 2015, to promote AfBPM. Mallinckrodt gave an internal presentation summarizing the movie as a story about the struggles of a pain patient looking for relief, who abuses OxyContin (a branded form of oxycodone produced by Purdue) and Percocet (a combination product containing oxycodone hydrochloride and acetaminophen) after a car crash (No Author, 2015c). In draft presentation slides from January 9, 2015, Mallnickrodt stated that the movie “provides the [Alliance for Balanced Pain Management] with [a] platform to comment and expand awareness of its mission,” and that it was an opportunity to “generate awareness of the Alliance for Balanced Pain Management as a credible information resource regarding appropriate, responsible pain management” (No Author, 2015c).

Green Room Communications also planned for AfBPM to issue a statement about the movie “CAKE” on January 23, 2015, the date of its widespread theatrical release following an initial smaller distribution (No Author, 2015c, 2015b). Phase 1 of the company’s planned approach involved developing a position on the movie, providing guidance to AfBPM members on how to post about it, and serving as a spokesperson for any media inquiries about AfBPM’s position (No Author, 2015c). Phase 2 involved “develop[ing] [an] op-ed to address realities of pain and the need for a balanced approach to pain management, including access and responsible use, prescribing, dispensing, storage and disposal” (No Author, 2015c). Mallinckrodt also sought to partner with one of the cast members and conduct a media tour with them (No Author, 2015c). The company ultimately chose Stacy Courtney (a stunt double for the lead actor, Jennifer Aniston), who was also a pain patient and the inspiration for the movie’s protagonist, Claire, believing that her involvement would be meaningful to people experiencing pain (No Author, 2015d). AfBPM ultimately published a blog post discussing the movie and used it “…to affirm the importance of access to integrated pain management and the need for safe and responsible prescribing, dispensing, using, storing, and disposing of medication to help reduce medicine abuse, misuse, and diversion” (Alliance for Balanced Pain Management, 2015; No Author, 2015b). However, we found no evidence that AfBPM conducted further promotions around the movie CAKE.

AfBPM transitioned from Mallinckrodt to the Alliance for Patient Access (AfPA) via an assignment agreement as of January 1, 2016 (No Author, 2016b). AfPA, as of August 2025, indicated that it was a non-profit organization made up of health care providers that advocate for policies to “support clinical decision making”, “limit abuse utilization management techniques”, and “acknowledg[e] each person’s individual health care needs” (“About”, no date). Mallinckrodt’s Director of Advocacy Relations, Kevin Webb, stated in an email to Deborah Pasko, director of medication safety and quality of the ASHP (an association of pharmacy professionals and the accrediting body for pharmacy residency and technician training programs)(No Author, n.d.), that the transition of the alliance to the AfPA would be a better long-term fit because the “AfPA provides an independent non-profit home for AfBPM…the degree of separation from Mallinckrodt will help ensure its activities are understood to be at the direction of its steering committee and not its donor(s)” (Webb, 2016b). Mallinckrodt stated it would continue to support AfBPM, but reduce its overall spending on the organization (Webb, 2016a, 2016b).

## Discussion

This study investigated the pharmaceutical industry’s approach to public outreach and issue framing through a coalition, documenting how Mallinckrodt established the Alliance for Balanced Pain Management in 2014 to advocate for what it called “balanced pain management.” Our review of internal industry documents revealed that Mallinckrodt created AfBPM to disseminate and legitimize its position that access to opioid medications needed to be maintained, that multimodal analgesia needed to be adopted, and that healthcare providers and patients shared responsibility for safeguarding medications to prevent misuse. The strategy incorporated multiple tactics outlined in the Policy Dystopia Model.

In previous research, the National Bureau of Economic Research reviewed industry documents drawn from litigation against Purdue Pharma to assess why there were slower increases in opioid overdoses in some states after the 1996 launch of OxyContin (oxycodone) (Alpert et al., 2019). At the time, several states still required the use of state-issued ‘triplicate prescription programs’ for Schedule II opioids, in which the prescriber, the patient, and the state each retained a paper copy of each prescription (Alpert et al., 2019). Purdue found that prescribers in triplicate-state jurisdictions had little interest in prescribing OxyContin, which led the company to market primarily to physicians in non-triplicate states (Alpert et al., 2019). Although triplicate programs were replaced with electronic prescribing, with the last program ending in 2004 (California), lower overdose rates in the triplicate states persisted through 2018 (Alpert et al., 2019). Our work builds on this research, which first identified how opioid industry documents can identify regulations that limit inappropriate marketing of addictive medications. Our findings suggest that identifying and exposing the scope of a corporation’s direct control over a coalition’s activities and messaging could prevent public health responses from being influenced by industry-favored approaches. Although the US has made policy changes that have partially addressed the opioid epidemic, in 2024, there were still over 50,000 deaths attributed to opioid overdoses, many of which involved prescription medications rather than illicit drugs (No Author, 2025).

This study found that an opioid company used multiple tactics outlined in the Policy Dystopia Model, suggesting cross-industry transferability of coalition-building, image improvement, and information-dissemination strategies (Ulucanlar et al., 2016). Similar to the alcohol and tobacco industry’s coalition-building practices, Mallinckrodt created the AfBPM and recruited reputable nonprofit organizations, trade groups, and other third parties to legitimize its messaging and policy objectives by associating them with respected institutions that did not share the company’s stigma. The use and formation of coalitions are tactics described in the model (Ulucanlar et al., 2016). For example, to prevent tax increase legislation, the tobacco industry laundered messaging through respected advocacy organizations and coalitions to advance social justice and regressivity arguments that it lacked the credibility to make (Campbell and Balbach, 2009; Wakefield et al., 2025). Mallinckrodt relied on recruiting existing entities, another coalition-formation tactic outlined by the Policy Dystopia Model, but did not fabricate, fund, or develop coalition partners, as the tobacco industry has in some instances (Fallin et al., 2014; Ulucanlar et al., 2016). The impact of the opioid crisis, the legitimate need for pharmaceutical products for public welfare, and the necessity of pain treatment may have reduced the stigma of partnering with Mallinckrodt and reduced obstacles to finding legitimate partners. In contrast, the tobacco industry is less appealing to third parties, given the harm caused by the tobacco industry’s products, and widespread knowledge of its public deception and interference in law and regulation.

Mallinckrodt also formed AfBPM and promoted its involvement in the coalition to make the company appear as an active and responsible industry actor, despite its contributions to the opioid crisis, reflecting image-rehabilitation efforts captured by the Policy Dystopia Model (Ulucanlar, Fooks and Gilmore, 2016). Mallinckrodt’s messaging, spread via coalition media outreach and initiatives such as its patient safety checklist, may have appeared more legitimate to the public because of its backing by respected coalition partners. Deployment of Mallinckrodt messaging via AfBPM minimized its overt control over coalition positions while giving them the appearance of broad institutional support. The coalition served as a vehicle for Mallinckrodt’s messaging on ‘balanced pain management’ that discouraged policies that would restrict opioid marketing, that failed to address the company’s problematic behavior, and that placed the onus of mitigation on consumers and healthcare providers. AfBPM promoted consumer and provider education, multimodal analgesia, and drug disposal pouches as solutions rather than addressing industry behavior. Coalition framing promoted opioid crisis mitigation while avoiding solutions that involved regulation of marketing and sales practices that contributed to the epidemic’s continuation. Like the alcohol and tobacco industry’s discursive strategy of claiming policies will have unintended effects on public health, the AfBPM occasionally portrayed restrictions on opioid dispensing as reducing access to opioids critical to pain relief for patients. Alcohol and tobacco industry discursive strategies rely on Mallinckrodt’s aims to develop a “news machine,” indicating that it planned to place these narratives widely in the public media to influence public opinion and the policy environment, aligning with the Policy Dystopia Model tactic of “amplifying” the industry’s information (Ulucanlar et al., 2016). Amplification of industry information injected it into professional as well as public discourse, which could have influenced perceptions of how to mitigate the opioid crisis.

Our findings are also relevant to the commercialization of other substances presented as therapeutic and safe to use, such as cannabis (Abrams, 2018; No Author, 2024a), benzodiazepines (Edinoff, Nix and Hollier, 2021), MDMA (Mitchell, Bogenschutz and Lilienstein, 2021), and methamphetamines (Hunt et al., 2006; Farah, 2019). Manufacturers of these products, which may be therapeutic or harmful, may have financial incentives to form coalitions to advocate for their products’ safety and recruit unwitting outside organizations to improve their public image. Mallinckrodt’s positioning of ‘access to medicines’ as a public problem and ‘overuse and addiction’ as individual responsibilities unrelated to corporate marketing recalls the tobacco, alcohol, and food industry’s positioning of smoking, alcohol misuse, and unhealthy eating and drinking as individual responsibilities(Thomas et al., 2016; Martino et al., 2017). Mallinckrodt’s proposed solution of promoting responsible prescribing, dispensing, use, storage, and disposal of pain medications through public outreach follows a long history of health-harming industries developing ultimately ineffective educational campaigns rather than supporting regulations that could better protect public health (Landman et al., 2002; van Schalkwyk et al., 2022). Traditionally, and in the case of Mallinckrodt, this approach relied on recruiting credible affiliates, establishing partnerships, and focusing educational messages on individual responsibility. Exposing the fact that corporate messaging routinely proposes that health problems caused by marketing unsafe products are individual responsibilities could help emphasize the social, political, and commercial factors that underlie public health problems, many of which are created by industry activities in pursuit of higher profits (Lacy-Nichols et al., 2022).

Our findings have limitations. An examination of the Policy Dystopia Model and its relevance to opioid industry behavior would be better suited to a study of Mallinckrodt’s overall efforts at the time of our investigation. The narrow focus of our study on Mallinckrodt’s outreach coalition inherently limited the tactics involved and may have missed other applicable Policy Dystopia Model strategies used by the company outside of the Alliance for Balanced Pain Management. Despite the narrow focus of our study, we determined that opioid industry messaging and interference tactics have reflected recurring alcohol and tobacco industry strategies. Future research should explore Mallinckrodt and other opioid manufacturers’ behaviors more broadly to determine the full applicability of the Policy Dystopia Model to opioid industry interference tactics.

## Conclusions

Mallinckrodt used the Alliance for Balanced Pain Management (AfBPM), a coalition it created and directed, to improve its image after the reputation of the pharmaceutical industry was compromised due to the US opioid crisis. Mallinckrodt’s Government Affairs department recruited non-industry stakeholders to promote the concept of balanced pain. The company also emphasized proper use and disposal of opioids to redirect attention from calls to regulate the widespread marketing of addictive medications. Mallinckrodt viewed these efforts as a strategy to portray itself as a responsible opioid manufacturer. Our findings suggest that additional efforts are needed to raise public awareness of the extent to which the pharmaceutical industry is involved in creating supposedly neutral coalitions that leverage professional organizations not part of the industry yet that reflect its preferred messaging. In addition, Mallinckrodt used the coalition it had created to promote other products it produced, such as Medsaway pouches (News Detail ∣ Mallinckrodt Pharmaceuticals, no date). Increased awareness of these outreach techniques and greater regulation of third-party advertising for pharmaceutical companies could help prevent future public health crises in which responses focus on individualized solutions while avoiding restrictions on industry activities and responsibilities.

## Supplementary Material

Supplement

The online version contains supplementary material available at https://doi.org/10.1057/s41599-026-08961-8.
