# Supplementary material for: Understanding pharmaceutical industry coalitions: a case study of Mallinckrodt
Source: Humanit Soc Sci Commun. Author manuscript; Available in PMC 2026 Oct 9. (PMC13649321; doi:10.1057/s41599-026-08961-8)
Supplement: Supplementary file 1 [file NIHMS2212123-supplement-Supplement.docx]

5,402,199 documents in UCSF - JHU Opioid Industry Documents Archive

6,635 documents identified in searches

6,502 non-relevant documents

134 relevant documents identified

25 documents included in manuscript

Excluded 109 documents of limited relevance
